# Surgical Management of Isolated Adrenal Metastases: A Retrospective Comparative Study of Open, Laparoscopic, and Robotic Adrenalectomy

**DOI:** 10.3390/jcm15082876

**Published:** 2026-04-10

**Authors:** Alessia Fassari, Angelo Iossa, Alessandra Micalizzi, Sara Giovampietro, Giulio Lelli, Daniele Crocetti, Claudio Letizia, Luigi Petramala, Antonio Carbone, Paolo Sapienza, Laurent Sulpice, Giuseppe Cavallaro

**Affiliations:** 1 General Surgery Unit, Centre Hospitalier de Rennes, 35000 Rennes, France; alessia.fassari@gmail.com (A.F.); laurent.sulpice@chu-rennes.fr (L.S.); 2 General Surgery Unit, ICOT Hospital, 04100 Latina, Italy; angelo.iossa@uniroma1.it (A.I.); alessandra.micalizzi@uniroma1.it (A.M.); saragiova91@gmail.com (S.G.); giuliolelli1987@gmail.com (G.L.); 3 Department of Surgery, Sapienza University, 00185 Rome, Italy; daniele.crocetti@uniroma1.it (D.C.); paolo.sapienza@uniroma1.it (P.S.); 4 Department of Clinical, Internal Medicine, Anesthesiology and Cardiovascular Sciences, Sapienza University, 00185 Rome, Italy; claudio.letizia@uniroma1.it; 5 Department of Translational and Precision Medicine, Sapienza University, 00175 Rome, Italy; luigi.petramala@uniroma1.it; 6 Department of Medico-Surgical Sciences and Biotechnologies, Sapienza University, 00185 Rome, Italy; antonio.carbone@uniroma1.it

**Keywords:** adrenal metastasis, adrenalectomy, robotic surgery

## Abstract

**Background**: Isolated adrenal metastases may occur in several solid malignancies, and surgical resection has been associated with improved local control and potential survival benefit in carefully selected patients. Open adrenalectomy has traditionally been considered the standard approach, while laparoscopic adrenalectomy has progressively gained acceptance as a minimally invasive alternative. More recently, robotic adrenalectomy has been introduced. However, its role in the management of adrenal metastases remains incompletely defined. **Methods:** This retrospective comparative study analyzed a prospectively maintained database including patients who underwent adrenalectomy for isolated adrenal metastasis between January 2001 and December 2025 at two academic centers. Patients were stratified according to surgical approach into open, laparoscopic, and robotic groups. Perioperative outcomes, postoperative morbidity, resection margin status, and oncological adequacy of the resection were compared among groups. **Results**: A total of 89 patients underwent adrenalectomy for isolated adrenal metastasis (robotic n = 27, laparoscopic n = 28, open n = 34). Metastasis size was larger in the robotic group compared with the laparoscopic group (51.4 vs. 44.2 mm, *p* = 0.043). Laparoscopy showed the shortest operative time, with respect to both robotic (*p* = 0.042) and open surgery (*p* = 0.045). Estimated blood loss was significantly higher in the open group (263 mL) than in the robotic (117 mL) and laparoscopic groups (106 mL) (*p* = 0.042 and 0.040, respectively). R0 resection rates were comparable across approaches (96%, 89%, and 94%, respectively). Hospital stay was shorter after both robotic and laparoscopic surgery with respect to open surgery (2.5–3.1 vs. 5.6 days, *p* = 0.043 and 0.046, respectively). **Conclusions**: Open, laparoscopic, and robotic adrenalectomy are all feasible surgical options for isolated adrenal metastases with acceptable perioperative outcomes. Robotic adrenalectomy may represent a potential extension of the applicability of minimally invasive surgery.

## 1. Introduction

Adrenal metastases represent a relatively frequent manifestation of systemic dissemination in several solid malignancies, most commonly lung cancer, renal cell carcinoma, melanoma, and colorectal cancer. Although adrenal involvement has traditionally been considered a marker of advanced disease, growing evidence indicates that surgical resection may provide meaningful local control and, in carefully selected patients with isolated or oligometastatic adrenal disease, may be associated with prolonged survival compared with non-surgical management [1,2,3]. In this context, adrenal metastases constitute a distinct clinical entity within adrenal surgery, marked by heterogeneous tumor biology, variable systemic disease control, and the need for individualized multidisciplinary decision-making.

Open adrenalectomy has historically represented the surgical approach of reference, particularly for large lesions or suspected local invasion [4]. More recently, minimally invasive techniques have been increasingly adopted, although their role in metastatic disease remains debated and strongly influenced by tumor size, anatomical complexity, and prior oncologic treatments [5]. Robotic surgery has been introduced as a potential evolution of minimally invasive adrenalectomy [6]. However, evidence supporting its use specifically in adrenal metastases remains limited.

Unlike benign adrenal disease or primary adrenal malignancies, secondary adrenal involvement is typically encountered in the setting of oligometastatic or previously treated systemic cancer, where surgical resection is considered with curative or consolidative intent only in highly selected patients. Consequently, surgical series focusing exclusively on adrenal metastasectomy remain scarce, and comparative analyses of surgical approaches are often extrapolated from mixed cohorts or from studies predominantly addressing benign pathology. To date, comparative studies have primarily focused on laparoscopic versus open or laparoscopic versus robotic adrenalectomy, often including heterogeneous indications and only a small subset of patients with adrenal metastases. Notably, no study has yet directly compared open, laparoscopic, and robotic adrenalectomy within a single cohort exclusively dedicated to metastatic adrenal disease.

The aim of the present study was therefore to provide a comparative analysis of robotic, laparoscopic, and open adrenalectomy specifically in patients undergoing surgery for adrenal metastases. Although limited by sample size, this dual-center experience evaluates perioperative outcomes, postoperative morbidity, and oncological adequacy of surgical resection across all three approaches, with particular emphasis on whether robotic assistance may extend the indications of minimally invasive surgery to patients with larger or more complex adrenal metastases while preserving oncological radicality.

## 2. Methods

### 2.1. Study Design and Population

This study is a retrospective analysis of a prospectively maintained database including patients who underwent adrenal surgery for primary or secondary adrenal tumors at two academic centers with long-standing scientific and clinical collaboration. Patients were treated between January 2001 and December 2025. Institutional Review Board (IRB) approval was obtained for the establishment and prospective maintenance of the database, which allowed the collection and scientific use of anonymized clinical data for research purposes.

All surgical procedures were performed according to standardized institutional protocols that have been established for many years and are consistent with current standards of perioperative management. Surgical procedures were performed by three senior staff surgeons, all of whom had extensive experience in adrenal surgery and minimally invasive techniques (laparoscopic and robotic approaches), each performing more than 30 adrenalectomies per year and more than 100 robotic procedures annually.

The choice of surgical approach (open, laparoscopic, or robotic) evolved progressively over the study period, moving from open surgery (particularly for suspected malignancy) to minimally invasive techniques. More recently, the indication for robotic surgery was also influenced by the availability of robotic platforms. In all cases, the final surgical strategy was determined at the time of surgery after multidisciplinary oncological evaluation and tumor board discussion.

Prior to surgery, all patients provided written informed consent, including consent for the use of anonymized clinical data for scientific evaluation and publication. Institutional Review Board (IRB) approval was obtained from the Human Research Ethics Committee of the Department of Surgery “Pietro Valdoni”, Sapienza University of Rome (Approval No. 1999/24/A, approved on 25 June 1999). This approval refers to the ethical authorization for the institutional adrenal surgery database from which the present study cohort was derived.

The study was conducted in accordance with the Declaration of Helsinki and registered in the Research Registry (researchregistry11787) [7].

### 2.2. Inclusion and Exclusion Criteria and Data Collection

All patients who underwent adrenalectomy for isolated adrenal metastasis, confirmed on final histopathology, were included in the study. Demographic, clinical, biochemical, imaging, surgical, and pathological data were extracted from the prospectively maintained database. All patients were evaluated and discussed by the institutional tumor board prior to the indication for surgery.

Exclusion criteria included patients undergoing adrenalectomy for primary adrenal tumors, patients with non-isolated adrenal metastases (i.e., presence of synchronous extra-adrenal metastatic disease), and patients with incomplete clinical or pathological data. Time to detection of adrenal metastasis was defined as the interval between the diagnosis of the primary tumor and the radiological diagnosis of adrenal metastatic disease.

### 2.3. Tumor Characteristics

Tumor-related variables included the primary tumor histology, laterality of the adrenal metastasis, and tumor size. Tumor size was defined as the maximum diameter of the adrenal metastasis measured on preoperative cross-sectional imaging (computed tomography or magnetic resonance imaging), using the largest axial dimension reported by the radiologist. In cases with multiple imaging studies, the most recent examination prior to surgery was considered for analysis.

Resection margin status was assessed on the final pathological examination and classified as radical (R0), defined as complete tumor removal with no microscopic residual disease, or non-radical (R1 or R2), indicating the presence of microscopic or macroscopic residual tumor, respectively.

### 2.4. Surgical Approach and Study Groups

Patients were retrospectively stratified according to surgical approach into three groups: Group A (ROB), including patients who underwent robotic adrenalectomy via a lateral transperitoneal approach; Group B (LAP), including patients who underwent laparoscopic adrenalectomy via a lateral transperitoneal approach; and Group C (OPEN), including patients who underwent open adrenalectomy through a midline or subcostal incision.

### 2.5. Surgical Technique

Open adrenalectomy was performed through a standard subcostal or midline incision, according to tumor laterality and surgeon preference. On the left side, colonic mobilization with splenopancreatic detachment was routinely performed to access the retroperitoneal space and allow identification and control of the left adrenal vein at its confluence with the left renal vein. On the right side, mobilization of the right colon and hepatic lobe was undertaken to expose the retrohepatic inferior vena cava and identify the right adrenal vein draining directly into the vena cava, as well as the right renal vein. In selected cases, limited duodenopancreatic mobilization was required to achieve adequate exposure of the vena cava.

Laparoscopic adrenalectomy was performed using the standard lateral transperitoneal approach. Patients were placed in a lateral decubitus position, allowing gravity-assisted retraction of intra-abdominal organs. Three trocars were placed in the left-sided procedures and four in right-sided procedures in the subcostal region. On the left side, gravity facilitated mobilization of the spleen and pancreatic tail, whereas on the right side it aided retraction of the right hepatic lobe, permitting safe access to the adrenal space and vascular pedicle.

**Figure 1:**
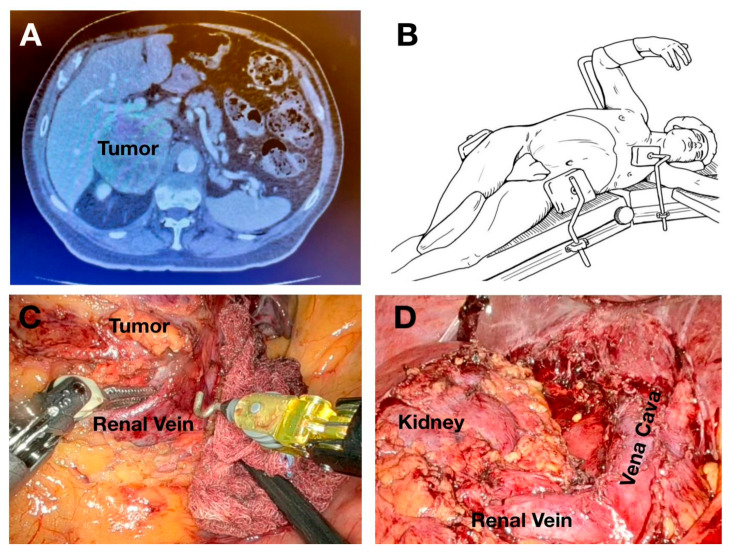
Right adrenal metastasis evidentiated on CT scan (**A**); patient position on the table for right robotic adrenalectomy (**B**); robotic dissection from surrounding structures (**C**), and final intraoperative view (**D**).

**Figure 2:**
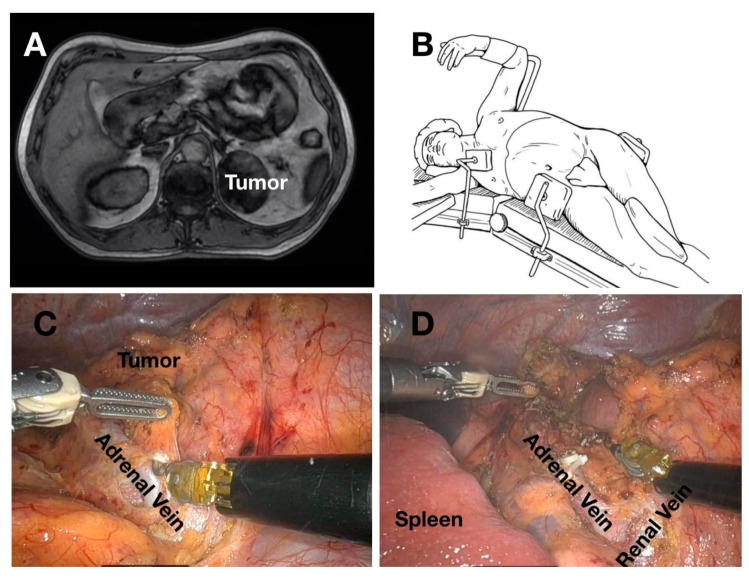
Left adrenal metastasis evidentiated on MRI (**A**); patient position on the table for left robotic adrenalectomy (**B**); robotic dissection from surrounding structures (**C**), and final intraoperative view (**D**).

Robotic adrenalectomy was conducted using the same lateral transperitoneal approach adopted for laparoscopy. Patients were positioned similarly, and trocar placement mirrored the laparoscopic configuration, with three robotic trocars plus one assistant laparoscopic trocar on the left side, and three robotic trocars plus two assistant trocars on the right side. Dissection of the adrenal gland and vascular control followed the same anatomical principles as in laparoscopy, with robotic articulation providing enhanced dexterity during vascular identification and adrenal vein control (Figure 1 and Figure 2).

All procedures were performed according to standardized adrenalectomy techniques routinely adopted at the participating institutions, consistent with previously described approaches in the literature.

### 2.6. Study Outcomes

The primary endpoint of the study was the comparison of perioperative outcomes among the three surgical approaches. Secondary endpoints included operative time, estimated intraoperative blood loss, conversion rate, postoperative complications, length of hospital stay, and oncological adequacy of the resection as assessed by resection margin status.

Perioperative variables included the surgical approach (open, laparoscopic, or robotic), the need for conversion to another approach and its underlying cause, operative time (defined as skin incision to skin closure, including docking time for the robotic group), estimated intraoperative blood loss, and resection margin status (R0 vs. R1/R2). Postoperative outcomes comprised length of hospital stay, defined as the number of days from surgery to discharge, and postoperative complications. All postoperative complications occurring within 30 days after surgery were recorded and graded according to the Clavien–Dindo classification. Both minor (grade I–II) and major (grade ≥III) complications were analyzed [8]. Intraoperative complications were documented separately and included vascular injuries or damage to adjacent organs requiring additional surgical maneuvers or conversion.

Mid- and long-term local complications were defined as surgery-related events occurring more than 30 days after adrenalectomy and involving the surgical site or adjacent anatomical structures. These included chronic postoperative pain, surgical site occurrences, incisional hernia, and local tumor recurrence.

### 2.7. Statistical Analysis

Continuous variables were tested for normality using the Shapiro–Wilk test. Variables with a normal distribution were expressed as mean ± standard deviation. Comparisons of continuous variables among the three groups were first performed using one-way analysis of variance (ANOVA). When a significant overall difference was identified, pairwise comparisons between groups were conducted using post hoc tests.

Categorical variables were reported as absolute numbers and percentages and compared using Fisher’s exact test as part of univariate analyses of the main clinical outcomes. A *p* value < 0.05 was considered statistically significant. All statistical analyses were performed using R statistical software (version 4.4.1; R Foundation for Statistical Computing, Vienna, Austria).

## 3. Results

### 3.1. Patient Characteristics and Oncological Features

The study included 89 patients undergoing adrenalectomy for metastatic disease, divided into robotic (ROB, n = 27), laparoscopic (LAP, n = 28), and open (OPEN, n = 34) approaches. Baseline demographic characteristics are summarized in Table 1 and were comparable among groups, with no significant differences in mean age, sex distribution, primary tumor type, side of metastasis, or time interval between primary tumor diagnosis and adrenal metastasis. Lung and renal carcinomas represented the most frequent primary tumors across all groups. The mean time from primary tumor diagnosis to adrenal metastasis was similar among the three approaches, suggesting comparable oncological timing of disease presentation.

Metastasis size differed significantly among groups. The robotic group showed larger lesions compared to the laparoscopic group (51.4 vs. 44.2 mm, *p* = 0.043), while no significant difference was observed between robotic and open surgery. Open surgery was more frequently used for larger metastases compared to laparoscopy (*p* = 0.041).

Baseline demographic characteristics and oncological features of patients undergoing adrenalectomy for isolated adrenal metastases, stratified by surgical approach: robotic, laparoscopic, and open. Variables include age, sex, primary tumor histology, time interval between primary tumor diagnosis and adrenal metastasis, laterality of metastasis, and metastatic lesion size on preoperative imaging. Continuous variables are reported as mean ± standard deviation unless otherwise specified. Comparisons of continuous variables were performed using one-way ANOVA, and categorical variables were compared using Fisher’s exact test.

### 3.2. Operative and Postoperative Outcomes

Operative and postoperative results are summarized in Table 2. Mean operative time was significantly different among the three approaches. Laparoscopy showed the shortest operative time, while robotic surgery required longer operative times than laparoscopy (*p* = 0.042) but remained significantly shorter than open surgery (*p* = 0.045), even when docking time was included in the robotic group.

Intraoperative blood loss was comparable between robotic and laparoscopic approaches, whereas open surgery was associated with significantly higher blood loss compared to both minimally invasive techniques (*p* = 0.042 vs. robotic and *p* = 0.040 vs. laparoscopy).

R0 resection rates were high and comparable across all groups (*p* = 0.52). Intraoperative complication rates did not differ significantly among the three approaches (*p* = 0.51). However, the nature of complications differed by approach, with vascular injuries being the main cause of conversion in the minimally invasive groups and splenic injuries occurring in the open group.

Postoperative complications occurred in 7 (25.9%), 9 (32.1%), and 13 (38.2%) patients in the robotic, laparoscopic, and open groups, respectively, without statistically significant differences among the approaches (*p* = 0.47). Major complications (Clavien–Dindo ≥ III) were rare and similarly distributed across groups (*p* = 0.29).

Mean postoperative hospital stay was significantly shorter after robotic and laparoscopic surgery compared to open adrenalectomy (2.5 vs. 3.1 vs. 5.6 days, *p* = 0.043 and *p* = 0.046 for robotic and laparoscopic vs. open), while no difference was observed between robotic and laparoscopic approaches.

Mid- and long-term local complications, including chronic pain, surgical site occurrences, incisional hernias, and local recurrences, were numerically lower after robotic surgery, although these differences did not reach statistical significance.

Mean follow-up duration was shorter in the robotic group compared to the open group, reflecting the more recent adoption of the robotic approach.

Operative details and postoperative outcomes of patients undergoing adrenalectomy for isolated adrenal metastases according to surgical approach. Variables include operative time (including docking time in the robotic group), estimated intraoperative blood loss, resection margin status, intraoperative and postoperative complications (graded according to the Clavien–Dindo classification), conversion to open surgery, length of postoperative hospital stay, mid- and long-term local complications, and follow-up duration. *p*-values represent univariate comparisons between groups performed using one-way ANOVA for continuous variables and Fisher’s exact test for categorical variables.

## 4. Discussion

The management of isolated adrenal metastases remains a complex and controversial challenge in surgical oncology. Although adrenal involvement has historically been interpreted as a manifestation of disseminated systemic disease, growing evidence indicates that surgical resection can result in significant oncological benefit in carefully selected patients with isolated or oligometastatic disease, especially when complete resection is possible. In a large multicenter retrospective cohort, Wachtel et al. demonstrated that adrenal metastasectomy is associated with prolonged overall survival, particularly in patients with isolated adrenal metastases undergoing R0 resection, while the presence of active extraadrenal disease at the time of surgery and positive resection margins were found to be independent prognostic factors for poor outcome [9].

These findings were further supported in a recent meta-analysis by Kong et al., who reported a pooled 5-year overall survival of approximately 38% after adrenalectomy for adrenal metastases, confirming the possibility of clinically relevant long-term survival in selected patients [10]. Overall, large retrospective series and systematic reviews thus support the role of surgery as a component of a multidisciplinary therapeutic strategy in carefully selected cases [11,12].

In this context, the choice of surgical approach represents a crucial technical and clinical consideration. Open adrenalectomy has long been considered the standard reference approach, particularly in the management of large adrenal lesions or in situations where strict oncological radicality and optimal vascular control are mandatory. However, open surgery is consistently associated with increased operative trauma, higher blood loss, and prolonged postoperative recovery, which may negatively affect perioperative outcomes and delay subsequent oncologic treatments [13]. The progressive diffusion of minimally invasive adrenalectomy, since the first laparoscopic adrenalectomy described by Gagner et al. in 1992, has consequently aimed to reduce surgical burden while preserving oncological principles [14]. Several multicenter experiences have demonstrated that a laparoscopic approach can be safely performed in patients with adrenal metastases when appropriate selection criteria are applied. A large comparative single-center study by Kwak et al. confirmed that laparoscopic adrenalectomy offers clear perioperative advantages over open surgery without compromising locoregional control or overall survival in selected patients [15]. Similarly, in a retrospective multicenter Japanese study, Goto et al. reported acceptable morbidity and meaningful long-term survival, showing that oncological outcomes are primarily driven by disease biology, particularly extra-adrenal disease and margin status, rather than by the minimally invasive approach itself [16]. Tumor size emerged as a key technical limitation, supporting a selective minimally invasive strategy and explaining why larger or anatomically complex metastases are still often managed with an open approach.

More recently, robotic surgery has emerged as a further step in the evolution of minimally invasive adrenalectomy. By combining advanced depth perception, enhanced instrument articulation, and optimized surgeon ergonomics, it may facilitate meticulous dissection and reliable vascular control in anatomically complex settings, particularly in close proximity to major vascular structures [17]. This evolution parallels what has been observed in other fields of complex oncologic surgery, such as pancreatic surgery, where robotic assistance has progressively expanded the applicability of minimally invasive approaches in technically challenging procedures, leading to a paradigm increasingly centered on a comparison between open and robotic surgery rather than conventional laparoscopy [18].

Piccoli et al., in a large single-center comparative series, showed that robotic adrenalectomy provides perioperative advantages over laparoscopy, particularly in technically demanding scenarios such as left-sided lesions, obese patients, and larger tumors, while maintaining comparable safety and oncological adequacy, supporting the role of the robotic platform as a facilitating tool rather than a change in surgical indication [19]. Similarly, a recent systematic review and meta-analysis by Zhang et al., focusing on large adrenal tumors including malignant and metastatic lesions, demonstrated that robotic adrenalectomy is associated with reduced blood loss, lower conversion rates, and shorter hospital stay compared with laparoscopy, without compromising perioperative safety, although at higher cost [20].

In the present study, robotic adrenalectomy was preferentially performed in patients with significantly larger adrenal metastases compared with laparoscopy, while achieving perioperative outcomes comparable to those observed with minimally invasive techniques. Despite longer operative times, robotic surgery was associated with limited intraoperative blood loss and a short postoperative hospital stay, approaching the results achieved with laparoscopy and clearly exceeding open surgery in terms of postoperative recovery. Taken together, these findings suggest that robotic assistance may potentially represent an extension of the applicability of minimally invasive surgery to selected patients with larger or more complex adrenal metastases, rather than a replacement of existing techniques. However, these findings should be interpreted cautiously given the retrospective design and the limited sample size of the present study.

The favorable perioperative outcomes observed after minimally invasive adrenalectomy should also be interpreted within the broader context of contemporary perioperative care. The increasing adoption of Enhanced Recovery After Surgery (ERAS) protocols has contributed to reducing postoperative morbidity and length of hospital stay after adrenal surgery, particularly when combined with minimally invasive approaches [21]. Reduced surgical trauma and lower intraoperative blood loss facilitate adherence to ERAS principles, potentially amplifying the recovery benefits observed after laparoscopic and robotic adrenalectomy. In this integrated setting, robotic assistance may further support optimized perioperative recovery in anatomically complex cases without compromising oncological adequacy.

Beyond perioperative recovery, oncological adequacy remains the central determinant of clinical benefit. In this setting, complete (R0) resection represents a prerequisite, rather than a variable influenced by surgical approach. Consistent with previous large multicenter analyses and the recent European Society of Endocrine Surgeons (ESES) consensus, the present findings confirm that the potential oncological benefit is primarily determined by appropriate patient selection within a multidisciplinary framework and the ability to achieve uncompromised tumor clearance, irrespective of the technique adopted [22]. In our series, high R0 resection rates were observed across robotic, laparoscopic, and open approaches. However, the study was not designed to evaluate differences in long-term oncological outcomes, which remain largely driven by underlying tumor biology.

Interpretation of survival after adrenal metastasectomy is further challenged by the marked heterogeneity related to primary tumor histology, with renal cell carcinoma typically associated with more favorable outcomes than colorectal, lung, or urothelial primaries. This histology-driven variability limits the attribution of survival differences to surgical strategy alone in single-center series and reinforces the central role of multidisciplinary decision-making [23,24]. In the present cohort, lung and renal cell carcinoma represented the most frequent primary tumors. However, the relatively small number of patients within each histological subgroup precluded meaningful comparative analysis according to tumor type. Finally, the morbidity associated with adrenal metastasectomy underscores that the potential benefit of surgery relies primarily on appropriate patient selection and oncological timing, rather than on surgical technique alone [25].

The present study has several limitations, including its retrospective design, potential selection bias related to surgical approach, and a limited sample size precluding robust multivariable adjustment. In particular, the relatively small number of events for key outcomes would make regression-based analyses statistically unstable and prone to overfitting. In addition, the follow-up duration was shorter in the robotic group, reflecting the more recent adoption of this technique and introducing a potential temporal bias that limits meaningful comparison of long-term outcomes. Finally, the heterogeneity of primary tumor histology further complicates the interpretation of oncological results, which were not the primary objective of the present analysis.

Despite these limitations, the present analysis provides a homogeneous cohort exclusively focused on isolated adrenal metastases and allows a comparative assessment of open, laparoscopic, and robotic adrenalectomy within this specific clinical setting. Future prospective studies and multicenter registries are needed to better define patient selection criteria, assess long-term oncological outcomes, and clarify the cost-effectiveness of the different surgical approaches in the management of adrenal metastatic disease. Until such data are available, the choice of surgical technique, including robotic assistance, should be individualized within experienced centers, based on tumor characteristics, patient factors, and institutional expertise within a multidisciplinary framework.

## 5. Conclusions

In this retrospective comparative study, open, laparoscopic, and robotic adrenalectomy for isolated adrenal metastases were all feasible surgical options with acceptable perioperative outcomes. Minimally invasive approaches were associated with shorter postoperative recovery compared with open surgery, while oncological adequacy was comparable across techniques. The robotic approach was more frequently used in patients with larger adrenal metastases without an apparent increase in perioperative morbidity and may suggest a potential extension of the applicability of minimally invasive surgery to selected complex cases. However, these findings should be considered exploratory and further prospective and multicenter studies are needed to better define the role of each surgical approach and optimize patient selection.

## Figures and Tables

**Table 1:** Patient characteristics and oncological features.

Variable	ROB (n = 27)	LAP (n = 28)	OPEN (n = 34)	*p* Value
Mean age (years) +/− SD	64.6 +/− 6.4	67.2 +/− 8.9	69.3 +/− 7.7	0.346
Sex (M/F)	12/15	15/13	18/16	0.75
Primary tumor				0.95
(1) lung	10	12	16	
(2) kidney	12	11	14	
(3) melanoma	2	4	5	
(4) colorectal	1	1	3	
Time to metastasis (months), mean ± SD	14.6 ± 2.8	16.4 ± 6.3	15.1 ± 8.2	A vs. B *p* = 0.143 A vs. C *p* = 0.227 B vs. C *p* = 0.304
Side (R/L)	12/16	14/13	19/15	NC
Metastasis size (mm), mean ± SD	51.4 ± 8.6	44.2 ± 7.7	55.8 ± 10.1	A vs. B *p* = 0.043 A vs. C *p* = 0.076 B vs. C *p* = 0.041

ROB, robotic adrenalectomy; LAP, laparoscopic adrenalectomy; OPEN, open adrenalectomy; R, right; L, left; SD, standard deviation; NS, not significant.

**Table 2:** Operative and postoperative outcomes.

Variable	ROB (n = 27)	LAP (n = 28)	OPEN (n = 34)	*p* Value
Operative time (min), mean ± SD *	77.4 ± 8.2	64.2 ± 7.3	83.5 ± 11.4	A vs. B *p* = 0.042 A vs. C *p* = 0.045 B vs. C *p* = 0.037
Intraoperative blood loss (mL), mean ± SD	117.4 ± 23.4	105.6 ± 18.5	263.2 ± 75.3	A vs. B *p* = 0.068 A vs. C *p* = 0.042 B vs. C *p* = 0.040
Resection margins				0.52
- R0	26	25	32	
- R1	1	2	2	
- R2	-	-	-	
Intraoperative complications, n	-	2 **	2 ***	0.51
Conversion to open surgery, n	-	3 ****	-	0.11
Postoperative complications, n	7	9	13	0.47
(1) I–II	7	7	10	
(2) III–IV	-	2	3	0.29
Length of stay (days), mean ± SD	2.5 ± 0.4	3.1 ± 0.4	5.6 ± 0.4	A vs. B *p* = 0.058 A vs. C *p* = 0.043 B vs. C *p* = 0.046
Mid and long-term local complications	2 ^	5 ^^	11 ^^^	NC
Follow-up (months), mean ± SD	38.2 ± 14.3	48.5 ± 11.3	78.6 ± 8.2	A vs. B *p* = 0.063 A vs. C *p* = 0.037 B vs. C *p* = 0.042

ROB, robotic adrenalectomy; LAP, laparoscopic adrenalectomy; OPEN, open adrenalectomy; SD, standard deviation; NS, not significant. * Operative time includes docking time in the robotic group. ** One right renal vein injury requiring conversion and vascular repair; one accessory hepatic vein injury requiring conversion and hemostasis by vein closure. *** Two splenectomies performed for intraoperative splenic injury. **** Conversions due to vascular injury (n = 2) and suspected lumbar muscle infiltration (n = 1). ^ One case of chronic subcostal pain and one trocar-site hernia. ^^ Two local recurrences (both R1 resections, 1 for melanoma metastasis, 1 for renal carcinoma metastasis), two trocar-site surgical site occurrences (SSO), and one trocar-site hernia. ^^^ Three cases of chronic subcostal pain, two local recurrences (both R1 resections, 1 for lung cancer metastasis, 1 for renal carcinoma metastasis), three surgical site occurrences (SSO), and three incisional hernias.

## Data Availability

The data presented in this study are available on request from the corresponding author.

## References

[1] Zheng Q.Y., Zhang G.H., Zhang Y., Guo Y.L. (2012). Adrenalectomy may increase survival of patients with adrenal metastases. Oncol. Lett..

[2] Samsel R., Cichocki A., Roszkowska-Purska K., Papierska L., Koalasińska-Ćwikła A., Karpeta E., Ostrowski T., Nowak K. (2020). Adrenal metastases—Long-term results of surgical treatment: Single-centre experience. Contemp Oncol..

[3] Lam K.Y., Lo C.Y. (2002). Metastatic tumours of the adrenal glands: A 30-year experience in a teaching hospital. Clin. Endocrinol..

[4] Uberoi J., Munver R. (2009). Surgical management of metastases to the adrenal gland: Open, laparoscopic, and ablative approaches. Curr. Urol. Rep..

[5] Quadri P., Esposito S., Coleoglou A., Danielson K.K., Masrur M., Giulianotti P.C. (2019). Robotic adrenalectomy: Are we expanding the indications of minimally invasive surgery?. J. Laparoendosc. Adv. Surg. Tech. A.

[6] Fassari A., Petramala L., Letizia C., Cavallaro G. (2024). Surgery for adrenocortical carcinoma: Do we have enough evidence to perform robotic approach? A systematic review. Indian J. Surg..

[7] World Medical Association (2013). World Medical Association Declaration of Helsinki: Ethical principles for medical research involving human subjects. JAMA.

[8] Dindo D., Demartines N., Clavien P.A. (2004). Classification of surgical complications: A new proposal with evaluation in a cohort of 6336 patients and results of a survey. Ann. Surg..

[9] Heather W., Beutner U., Bockhorn M., Matthew N.A., Ali T., Sareh P., Richard H.A., Douglas F.L., Benjamin J.C., Azadeh C.A. (2021). Adrenalectomy for secondary malignancy: Patients, outcomes, and indications. Ann. Surg..

[10] Kong J., Odisho T., Alhajahjeh A., Maqsood H.A., Al-Share B.A., Shahait M., Abubaker A., Kim S., Shahait A. (2024). Long-term survival following adrenalectomy for secondary adrenal tumors: A systematic review and meta-analysis. Am. J. Surg..

[11] Metman M.J.H., Viëtor C.L., Seinen A.J., Berends A.M.A., Hemmer P.H.J., Kerstens M.N., Feelders R.A., Franssen G.J.H., van Ginhoven T.M., Kruijff S. (2022). Outcomes after surgical treatment of metastatic disease in the adrenal gland: Valuable for the patient?. Cancers.

[12] Wachtel H., Dickson P., Fisher S.B., Kiernan C.M., Solórzano C.C. (2023). Adrenal metastasectomy in the era of immuno- and targeted therapy. Ann. Surg. Oncol..

[13] Zubair A.B., Arif M.H., Razzaq M.T., Zaman M., Haider Z., Fajar I.-E., Saleem S., Khalil A., Sabir M., Kaneez M. (2022). The spectrum of postoperative complications and outcomes after open adrenalectomy: An experience from a developing country. Cureus.

[14] Gagner M., Lacroix A., Bolté E. (1992). Laparoscopic adrenalectomy in Cushing’s syndrome and pheochromocytoma. N. Engl. J. Med..

[15] Kwak J., Bae H.L., Jung Y., Choi J., Hwang H., Kim J.H., Kim S.-J., Lee K.E. (2024). Comparative outcomes and prognostic indicators in adrenalectomy for adrenal metastasis. Surg. Endosc..

[16] Goto T., Inoue T., Kobayashi T., Yamasaki T., Ishitoya S., Segawa T., Ito N., Shichiri Y., Okumura K., Okuno H. (2020). Feasibility of laparoscopic adrenalectomy for metastatic adrenal tumors in selected patients: A retrospective multicenter study of Japanese populations. Int. J. Clin. Oncol..

[17] Piramide F., Bravi C.A., Paciotti M., Sarchi L., Nocera L., Piro A., Lores M.P., Balestrazzi E., Mottaran A., Farinha R. (2023). Robot-assisted adrenalectomy: Step-by-step technique and surgical outcomes at a high-volume robotic center. Asian J. Urol..

[18] Ferrari D., Violante T., Novelli M., Starlinger P.P., Smoot R.L., Reisenauer J.S., Larson D.W. (2024). The death of laparoscopy. Surg. Endosc..

[19] Piccoli M., Pecchini F., Serra F., Nigro C., Colli G., Gozzo D., Zirilli L., Madeo B., Rochira V., Mullineris B. (2021). Robotic versus laparoscopic adrenalectomy: Pluriannual experience in a high-volume center evaluating indications and results. J. Laparoendosc. Adv. Surg. Tech. A.

[20] Zhang S., Chen C., Mo C., Pei Z., Dong Z., Ning Z., Hou Z., Ding H. (2025). Robotic-assisted versus laparoscopic adrenalectomy for large adrenal tumors: A systematic review and meta-analysis. Int. Urol. Nephrol..

[21] Lelli G., Micalizzi A., Iossa A., Fassari A., Concistre A., Circosta F., Petramala L., De Angelis F., Letizia C., Cavallaro G. (2024). Application of enhanced recovery after surgery (ERAS) protocols in adrenal surgery: A retrospective, preliminary analysis. J. Minimal Access Surg..

[22] Mihai R., De Crea C., Guerin C., Torresan F., Agcaoglu O., Simescu R., Walz M.K. (2024). Surgery for advanced adrenal malignant disease: Recommendations based on European Society of Endocrine Surgeons consensus meeting. Br. J. Surg..

[23] Ferriero M., Iannuzzi A., Bove A.M., Tuderti G., Anceschi U., Misuraca L., Brassetti A., Mastroianni R., Guaglianone S., Leonardo C. (2024). Adrenalectomy for metastasis: The impact of primary histology on survival outcome. Cancers.

[24] Goujon A., Schoentgen N., Betari R., Thoulouzan M., Vanalderwerelt V., Oumakhlouf S., Brichart N., Pradere B., Roumiguie M., Rammal A. (2020). Prognostic factors after adrenalectomy for adrenal metastasis. Int. Urol. Nephrol..

[25] Bradley C.T., Strong V.E. (2014). Surgical management of adrenal metastases. J. Surg. Oncol..

